# Diosgenone: a second *P*2_1_ polymorph

**DOI:** 10.1107/S160053681202908X

**Published:** 2012-07-07

**Authors:** María-Guadalupe Hernández Linares, Gabriel Guerrero-Luna, Sylvain Bernès, Marcos Flores-Alamo, María A. Fernández-Herrera

**Affiliations:** a Escuelas de Ingeniería en Petróleos e Ingeniería Química, Universidad del Istmo, Ciudad Universitaria s/n, Sto. Domingo Tehuantepec, Oax. 70760, Mexico; b DEP Facultad de Ciencias Químicas, UANL, Guerrero y Progreso S/N, Col. Treviño, 64570 Monterrey, NL, Mexico; c Facultad de Química, Universidad Nacional Autónoma de México, México DF 04510, Mexico

## Abstract

Diosgenone [(20*S*,22*R*,25*R*)-spirost-4-en-3-one, C_27_H_40_O_3_] has been proposed as a new therapeutic alternative for the treatment of malaria. The first X-ray structure report for diosgenone was by Piro *et al.* [(2002). *Z. Naturforsch. Teil C*, **57**, 947–950] in the space group *P*2_1_ (*Z*′ = 2). We now report a new polymorph in the same space group, with two mol­ecules in the asymmetric unit. Both mol­ecules have similar conformations, characterized by a skewed envelope *A* ring, which contains the C=C bond conjugated with the ketone functionality at C3. The dimorphism results from a modification of the relative orientation of the mol­ecules in the asymmetric unit: two independent mol­ecules were arranged anti­parallel in the Piro report, while they are parallel in the present determination.

## Related literature

For the potential application of diosgenone as an anti­malarial drug, see: Saez *et al.* (1998 ▶); Echeverri *et al.* (2001 ▶). For a biotransformation of diosgenone, see: Wang *et al.* (2007 ▶, 2009 ▶). For the synthesis of diosgenone, see: Hunter & Priest (2006 ▶). For the structure of a monoclinic polymorph of diosgenone, see: Piro *et al.* (2002 ▶).
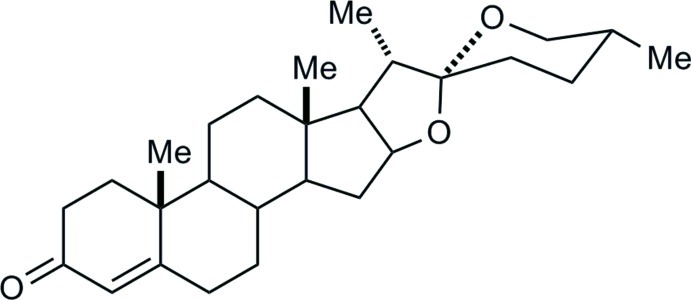


## Experimental

### 

#### Crystal data

C_27_H_40_O_3_
*M* *_r_* = 412.59
Monoclinic, 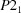

*a* = 10.3396 (6) Å
*b* = 7.6466 (4) Å
*c* = 29.9511 (17) Å
β = 97.207 (5)°
*V* = 2349.3 (2) Å^3^
*Z* = 4
Mo *K*α radiation
μ = 0.07 mm^−1^
*T* = 136 K
0.44 × 0.19 × 0.05 mm

#### Data collection

Oxford Diffraction Xcalibur (Atlas, Gemini) diffractometer
Absorption correction: analytical [*CrysAlis PRO* (Oxford Diffraction, 2009 ▶); based on expressions derived by Clark & Reid (1995 ▶)] *T*_min_ = 0.984, *T*_max_ = 0.996
11305 measured reflections
5346 independent reflections
3908 reflections with *I* > 2σ(*I*)
*R*_int_ = 0.042

#### Refinement

*R*[*F* ^2^ > 2σ(*F* ^2^)] = 0.052
*wR*(*F* ^2^) = 0.099
*S* = 1.03
5346 reflections
549 parameters
1 restraint
H-atom parameters constrained
Δρ_max_ = 0.23 e Å^−3^
Δρ_min_ = −0.19 e Å^−3^

### 

Data collection: *CrysAlis CCD* (Oxford Diffraction, 2009 ▶); cell refinement: *CrysAlis CCD*; data reduction: *CrysAlis RED* (Oxford Diffraction, 2009 ▶); program(s) used to solve structure: *SHELXS97* (Sheldrick, 2008 ▶); program(s) used to refine structure: *SHELXL97* (Sheldrick, 2008 ▶); molecular graphics: *SHELXTL* (Sheldrick, 2008 ▶) and *Mercury* (Macrae *et al.*, 2008 ▶); software used to prepare material for publication: *SHELXTL*.

## Supplementary Material

Crystal structure: contains datablock(s) I, global. DOI: 10.1107/S160053681202908X/gg2086sup1.cif

Structure factors: contains datablock(s) I. DOI: 10.1107/S160053681202908X/gg2086Isup2.hkl

Additional supplementary materials:  crystallographic information; 3D view; checkCIF report

## Figures and Tables

**Fig. 1.**
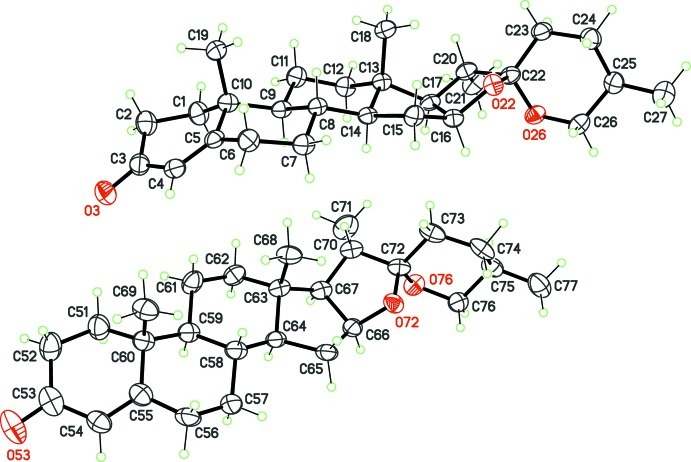
The asymmetric unit of the title compound. Displacement ellipsoids for non-H atoms are drawn at the 50% probability level.

**Fig. 2.**
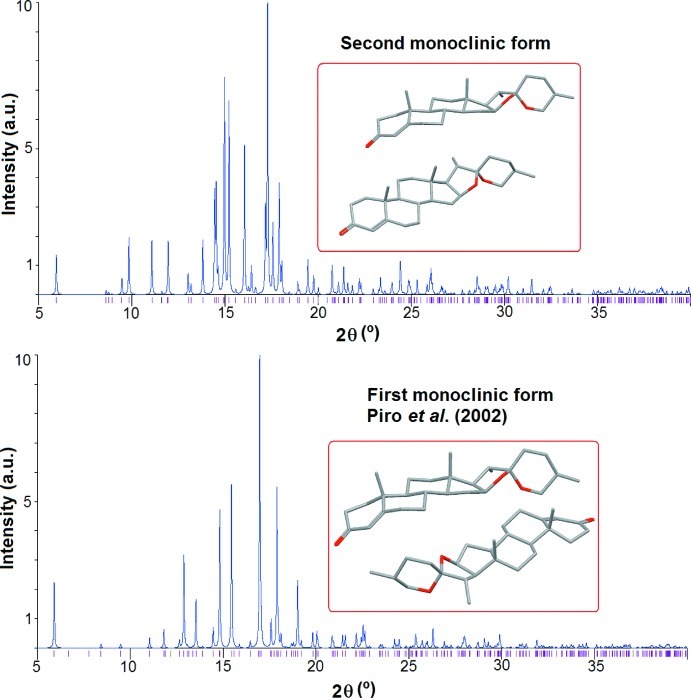
Simulated powder diffraction patterns for both P21 polymorphs of the title compound. Spectra were calculated with Mercury (Macrae et al., 2008) with λ = 1.5418 Å, by steps of 0.002° and peak shape defined by a FWHM of 0.05° in 2θ. The insets for each polymorph represent the asymmetric unit for the crystal, omitting H atoms for clarity. The asymmetric unit for the previously reported form (Piro et al., 2002) has been regrouped in order to be comparable to the unit used for the refinement of the new polymorph.

## supplementary crystallographic information

### Comment

A natural source of diosgenone is *Solanun nudum* (Saez *et al.*, 1998), present in different regions of South America. This steroid is also synthesized by oxidation of diosgenin, for instance, through the Swern oxidation. We obtained it using a modified Jones oxidation described in the literature (Hunter & Priest, 2006). The interest in diosgenone is currently growing, since it has been proposed by a group working in Colombia as a new therapeutic alternative for the treatment of malaria (Echeverri *et al.*, 2001; Saez *et al.*, 1998). This claim is in line with the fact that *Solanun nudum* is used by the community of Tumaco (Narino, Colombia) as a cure for malaria. A more academic interest is related to the biotransformation of diosgenone to isonuatigenone (C25-hydroxylation), which rearranges in acid media into nuatigenone, a rare nuatigenin-type steroid (Wang *et al.*, 2007, 2009). These studies allow a postulate for a new pathway of diosgenin metabolism.

The X-ray structure for diosgenone was described in space group *P*2_1_ (Piro *et al.*, 2002; CSD refcode: LUKXAQ). We have now discovered that a second polymorph in the same space group may be obtained if the crystallization is carried out by slow evaporation of an AcOEt/acetone (4:1) solution, while Piro *et al.* crystallized diosgenone from an ethanolic solution. The asymmetric unit, as in the previous report, contains two independent molecules (Fig. 1), with very similar conformations. The r.m.s. deviation for the fitted molecules is less than 0.2 Å. No significant conformational modification is observed by comparing molecules in both polymorphs: calculated r.m.s. deviations for pairs of molecules taken in different crystal forms are in the range 0.17 to 0.32 Å, the largest deviations being for the methyl group bonded to C25 in the *F* ring. However, simulated X-ray powder patterns for each form (Fig. 2) show clearly that these crystal forms are dimorphic. The crystal modification should thus be due to a reorientation of independent molecules in the asymmetric unit. In the case of the previously described structure (Piro *et al.*, 2002), the asymmetric unit may be described with two molecules arranged in such a way that the *A*→*F* rings sequence of one molecule is oriented antiparallel to the *A*→*F* sequence of the other one. In contrast, the asymmetric unit of the title polymorph described herein includes two parallel molecules (see insets in Fig. 2).

### Experimental

The title steroid was synthesized from diosgenin using a Jones oxidation described previously (Hunter & Priest, 2006). Diosgenine (2 g, 4.8 mmol) was dissolved in a CH_2_Cl_2_/acetone mixture (40 and 132 ml) and this solution was cooled to 263 K. Under stirring, the Jones reagent was added slowly, over 10 min., maintaining the temperature below 283 K. After addition, the mixture was further stirred at room temperature, until the color turned from orange to green. 2-Propanol was then added in order to eliminate the unreacted Jones reagent, and the product was extracted with AcOEt, washed with water, and dried over Na_2_SO_4_. The crude product was purified by chromatography on silica gel (AcOEt/hexane, 1:9 *v*/*v*), affording the title steroid (yield: 20%) and the Δ^4^-3,6 dione derivative. ^13^C-NMR for diosgenone: δ = 36.6 (C-1), 33.9 (C-2), 199.5 (C-3), 123.8 (C-4), 171.1 (C-5), 32.8 (C-6), 32.1 (C-7), 35.1 (C-8), 53.7 (C-9), 38.6 (C-10), 20.8 (C-11), 39.6 (C-12), 40.3 (C-13), 55.6 (C-14), 31.6 (C-15), 80.6 (C-16), 61.9 (C-17), 17.3 (C-18), 16.3 (C-19), 41.6 (C-20), 14.5 (C-21), 109.2 (C-22), 31.3 (C-23), 28.7 (C-24), 30.2 (C-25), 66.8 (C-26), 17.1 (C-27). Suitable single crystals were obtained by slow evaporation of an AcOEt/acetone (8:2) solution.

### Refinement

All H atoms were placed in idealized positions and refined as riding on their carrier atoms. Isotropic displacement parameters were calculated as *U*_iso_(H) = *xU*_eq_(carrier atom) where *x* = 1.5 for methyl H atoms and *x* = 1.2 otherwise. Absolute configuration was assigned from chiral centers with known configuration in the steroidal nucleus, and measured Friedel pairs (3382) were merged.

### Crystal data

**Table:**

C_27_H_40_O_3_	*F*(000) = 904
*M**_r_* = 412.59	*D*_x_ = 1.167 Mg m^−^^3^
Monoclinic, *P*2_1_	Melting point: 432 K
Hall symbol: P 2yb	Mo *K*α radiation, λ = 0.71073 Å
*a* = 10.3396 (6) Å	Cell parameters from 2248 reflections
*b* = 7.6466 (4) Å	θ = 3.4–26.0°
*c* = 29.9511 (17) Å	µ = 0.07 mm^−^^1^
β = 97.207 (5)°	*T* = 136 K
*V* = 2349.3 (2) Å^3^	Prism, colourless
*Z* = 4	0.44 × 0.19 × 0.05 mm

### Data collection

**Table:**

Oxford Diffraction Xcalibur (Atlas, Gemini) diffractometer	5346 independent reflections
Radiation source: Enhance (Mo) X-ray Source	3908 reflections with *I* > 2σ(*I*)
Graphite monochromator	*R*_int_ = 0.042
Detector resolution: 10.4685 pixels mm^-1^	θ_max_ = 26.7°, θ_min_ = 3.4°
ω scans	*h* = −13→9
Absorption correction: analytical [*CrysAlis PRO* (Oxford Diffraction, 2009); based on expressions derived by Clark & Reid (1995)]	*k* = −9→9
*T*_min_ = 0.984, *T*_max_ = 0.996	*l* = −37→34
11305 measured reflections	

### Refinement

**Table:**

Refinement on *F*^2^	Primary atom site location: structure-invariant direct methods
Least-squares matrix: full	Secondary atom site location: difference Fourier map
*R*[*F*^2^ > 2σ(*F*^2^)] = 0.052	Hydrogen site location: inferred from neighbouring sites
*wR*(*F*^2^) = 0.099	H-atom parameters constrained
*S* = 1.03	*w* = 1/[σ^2^(*F*_o_^2^) + (0.0356*P*)^2^ + 0.2215*P*] where *P* = (*F*_o_^2^ + 2*F*_c_^2^)/3
5346 reflections	(Δ/σ)_max_ = 0.001
549 parameters	Δρ_max_ = 0.23 e Å^−^^3^
1 restraint	Δρ_min_ = −0.19 e Å^−^^3^
0 constraints	

### Fractional atomic coordinates and isotropic or equivalent isotropic displacement parameters (Å^2^)

**Table:**

	*x*	*y*	*z*	*U*_iso_*/*U*_eq_	
C1	0.5754 (3)	0.1116 (4)	0.69355 (10)	0.0372 (8)	
H1A	0.5868	0.0139	0.7154	0.045*	
H1B	0.4900	0.1666	0.6960	0.045*	
C2	0.5743 (3)	0.0383 (4)	0.64620 (10)	0.0406 (8)	
H2A	0.6559	−0.0275	0.6443	0.049*	
H2B	0.5003	−0.0439	0.6397	0.049*	
C3	0.5620 (3)	0.1816 (4)	0.61200 (11)	0.0368 (8)	
O3	0.5019 (2)	0.1613 (3)	0.57407 (8)	0.0489 (6)	
C4	0.6307 (3)	0.3437 (4)	0.62540 (11)	0.0339 (7)	
H4A	0.6329	0.4333	0.6035	0.041*	
C5	0.6909 (3)	0.3724 (4)	0.66703 (10)	0.0283 (7)	
C6	0.7742 (3)	0.5326 (4)	0.67710 (10)	0.0341 (7)	
H6A	0.7613	0.6125	0.6509	0.041*	
H6B	0.8671	0.4974	0.6816	0.041*	
C7	0.7426 (3)	0.6299 (4)	0.71874 (10)	0.0319 (7)	
H7A	0.6565	0.6871	0.7119	0.038*	
H7B	0.8085	0.7228	0.7263	0.038*	
C8	0.7402 (3)	0.5111 (4)	0.75955 (9)	0.0251 (6)	
H8A	0.8305	0.4669	0.7691	0.030*	
C9	0.6494 (3)	0.3532 (4)	0.74742 (10)	0.0274 (7)	
H9A	0.5608	0.4036	0.7382	0.033*	
C10	0.6836 (3)	0.2473 (4)	0.70624 (10)	0.0291 (7)	
C11	0.6363 (3)	0.2383 (4)	0.78883 (10)	0.0337 (8)	
H11A	0.5728	0.1436	0.7801	0.040*	
H11B	0.7215	0.1832	0.7991	0.040*	
C12	0.5917 (3)	0.3425 (4)	0.82764 (10)	0.0309 (7)	
H12A	0.5026	0.3880	0.8185	0.037*	
H12B	0.5886	0.2646	0.8539	0.037*	
C13	0.6838 (3)	0.4950 (4)	0.84101 (9)	0.0261 (7)	
C14	0.6932 (3)	0.6075 (4)	0.79873 (10)	0.0253 (7)	
H14A	0.6020	0.6450	0.7880	0.030*	
C15	0.7635 (3)	0.7722 (4)	0.81842 (10)	0.0302 (7)	
H15A	0.8581	0.7510	0.8264	0.036*	
H15B	0.7508	0.8716	0.7971	0.036*	
C16	0.6966 (3)	0.8054 (4)	0.86036 (10)	0.0294 (7)	
H16A	0.6302	0.9004	0.8544	0.035*	
C17	0.6305 (3)	0.6331 (4)	0.87178 (10)	0.0263 (7)	
H17A	0.5342	0.6444	0.8633	0.032*	
C18	0.8184 (3)	0.4264 (4)	0.86118 (10)	0.0326 (7)	
H18A	0.8629	0.3752	0.8373	0.049*	
H18B	0.8075	0.3370	0.8839	0.049*	
H18C	0.8705	0.5232	0.8753	0.049*	
C19	0.8168 (3)	0.1547 (4)	0.71607 (10)	0.0339 (7)	
H19A	0.8844	0.2412	0.7258	0.051*	
H19B	0.8376	0.0964	0.6887	0.051*	
H19C	0.8130	0.0676	0.7399	0.051*	
C20	0.6594 (3)	0.6163 (4)	0.92306 (10)	0.0313 (7)	
H20A	0.7254	0.5214	0.9299	0.038*	
C21	0.5398 (3)	0.5704 (4)	0.94613 (11)	0.0421 (9)	
H21A	0.5074	0.4546	0.9361	0.063*	
H21B	0.4714	0.6577	0.9382	0.063*	
H21C	0.5640	0.5695	0.9788	0.063*	
C22	0.7243 (3)	0.7909 (4)	0.93750 (10)	0.0290 (7)	
O22	0.78615 (18)	0.8456 (3)	0.90003 (6)	0.0311 (5)	
C23	0.8256 (3)	0.7808 (4)	0.97853 (10)	0.0339 (8)	
H23A	0.9006	0.7109	0.9711	0.041*	
H23B	0.7877	0.7195	1.0029	0.041*	
C24	0.8740 (3)	0.9597 (4)	0.99535 (11)	0.0367 (8)	
H24A	0.9255	1.0138	0.9733	0.044*	
H24B	0.9313	0.9470	1.0242	0.044*	
C25	0.7585 (3)	1.0770 (4)	1.00196 (10)	0.0340 (8)	
H25A	0.7112	1.0230	1.0257	0.041*	
C26	0.6669 (3)	1.0798 (4)	0.95837 (11)	0.0359 (8)	
H26A	0.5909	1.1546	0.9622	0.043*	
H26B	0.7124	1.1321	0.9344	0.043*	
O26	0.62185 (19)	0.9090 (3)	0.94460 (7)	0.0328 (5)	
C27	0.7991 (4)	1.2607 (5)	1.01715 (12)	0.0480 (9)	
H27A	0.7217	1.3284	1.0221	0.072*	
H27B	0.8430	1.3176	0.9939	0.072*	
H27C	0.8588	1.2545	1.0452	0.072*	
C51	0.2234 (4)	0.4065 (5)	0.50640 (11)	0.0479 (9)	
H51A	0.2770	0.3160	0.5236	0.058*	
H51B	0.1309	0.3823	0.5094	0.058*	
C52	0.2432 (4)	0.3930 (5)	0.45691 (12)	0.0513 (10)	
H52A	0.3374	0.4016	0.4542	0.062*	
H52B	0.2122	0.2773	0.4452	0.062*	
C53	0.1716 (3)	0.5336 (5)	0.42910 (12)	0.0475 (9)	
O53	0.1312 (2)	0.5121 (4)	0.38911 (8)	0.0611 (8)	
C54	0.1587 (3)	0.7000 (5)	0.45163 (11)	0.0421 (9)	
H54A	0.1151	0.7927	0.4348	0.051*	
C55	0.2052 (3)	0.7301 (5)	0.49495 (11)	0.0386 (8)	
C56	0.2100 (4)	0.9128 (5)	0.51395 (11)	0.0471 (9)	
H56A	0.1587	0.9914	0.4922	0.056*	
H56B	0.3015	0.9542	0.5177	0.056*	
C57	0.1576 (4)	0.9241 (5)	0.55872 (11)	0.0462 (9)	
H57A	0.0622	0.9047	0.5540	0.055*	
H57B	0.1734	1.0432	0.5712	0.055*	
C58	0.2201 (3)	0.7912 (4)	0.59259 (11)	0.0351 (8)	
H58A	0.3158	0.8158	0.5984	0.042*	
C59	0.2006 (3)	0.6060 (4)	0.57195 (11)	0.0345 (8)	
H59A	0.1044	0.5920	0.5639	0.041*	
C60	0.2604 (3)	0.5871 (4)	0.52681 (11)	0.0354 (8)	
C61	0.2428 (4)	0.4588 (4)	0.60576 (11)	0.0426 (9)	
H61A	0.2140	0.3452	0.5921	0.051*	
H61B	0.3391	0.4569	0.6117	0.051*	
C62	0.1873 (3)	0.4792 (4)	0.65045 (11)	0.0402 (8)	
H62A	0.0915	0.4641	0.6454	0.048*	
H62B	0.2238	0.3870	0.6715	0.048*	
C63	0.2195 (3)	0.6582 (4)	0.67138 (11)	0.0307 (7)	
C64	0.1640 (3)	0.7983 (4)	0.63715 (10)	0.0318 (7)	
H64A	0.0688	0.7727	0.6300	0.038*	
C65	0.1743 (3)	0.9677 (4)	0.66473 (10)	0.0383 (8)	
H65A	0.2647	1.0133	0.6687	0.046*	
H65B	0.1149	1.0588	0.6504	0.046*	
C66	0.1328 (3)	0.9077 (4)	0.70958 (11)	0.0349 (8)	
H66A	0.0402	0.9414	0.7114	0.042*	
C67	0.1481 (3)	0.7065 (4)	0.71224 (11)	0.0333 (8)	
H67A	0.0595	0.6518	0.7082	0.040*	
C68	0.3673 (3)	0.6767 (5)	0.68339 (11)	0.0392 (8)	
H68A	0.4007	0.5776	0.7021	0.059*	
H68B	0.3868	0.7859	0.7000	0.059*	
H68C	0.4089	0.6783	0.6557	0.059*	
C69	0.4108 (3)	0.6055 (5)	0.53395 (11)	0.0443 (9)	
H69A	0.4436	0.6093	0.5047	0.066*	
H69B	0.4490	0.5052	0.5512	0.066*	
H69C	0.4349	0.7136	0.5505	0.066*	
C70	0.2126 (3)	0.6711 (4)	0.76027 (11)	0.0357 (8)	
H70A	0.3058	0.6409	0.7584	0.043*	
C71	0.1534 (4)	0.5204 (5)	0.78430 (13)	0.0572 (11)	
H71A	0.1959	0.5130	0.8154	0.086*	
H71B	0.1665	0.4106	0.7686	0.086*	
H71C	0.0599	0.5409	0.7843	0.086*	
C72	0.2107 (3)	0.8498 (4)	0.78344 (11)	0.0325 (7)	
O72	0.2163 (2)	0.9736 (3)	0.74834 (7)	0.0345 (5)	
C73	0.3239 (3)	0.8823 (5)	0.81953 (11)	0.0421 (9)	
H73A	0.4047	0.8930	0.8052	0.051*	
H73B	0.3339	0.7799	0.8399	0.051*	
C74	0.3084 (3)	1.0466 (5)	0.84749 (11)	0.0442 (9)	
H74A	0.3787	1.0510	0.8731	0.053*	
H74B	0.3158	1.1518	0.8287	0.053*	
C75	0.1776 (3)	1.0458 (5)	0.86483 (10)	0.0397 (8)	
H75A	0.1745	0.9421	0.8850	0.048*	
C76	0.0728 (3)	1.0224 (4)	0.82481 (11)	0.0367 (8)	
H76A	−0.0139	1.0226	0.8355	0.044*	
H76B	0.0761	1.1223	0.8040	0.044*	
O76	0.08903 (19)	0.8629 (3)	0.80108 (7)	0.0373 (5)	
C77	0.1522 (4)	1.2083 (5)	0.89150 (12)	0.0548 (10)	
H77A	0.2242	1.2256	0.9156	0.082*	
H77B	0.0706	1.1940	0.9046	0.082*	
H77C	0.1453	1.3102	0.8715	0.082*	

### Atomic displacement parameters (Å^2^)

**Table:**

	*U*^11^	*U*^22^	*U*^33^	*U*^12^	*U*^13^	*U*^23^
C1	0.0408 (19)	0.0340 (18)	0.0368 (19)	−0.0103 (16)	0.0045 (15)	−0.0027 (16)
C2	0.0393 (19)	0.0381 (19)	0.044 (2)	−0.0111 (16)	0.0023 (16)	−0.0079 (17)
C3	0.0295 (17)	0.043 (2)	0.037 (2)	0.0026 (15)	0.0015 (15)	−0.0086 (17)
O3	0.0464 (14)	0.0565 (16)	0.0406 (14)	0.0030 (13)	−0.0077 (12)	−0.0125 (13)
C4	0.0335 (17)	0.0310 (17)	0.0366 (19)	0.0048 (15)	0.0017 (15)	0.0014 (16)
C5	0.0225 (15)	0.0267 (16)	0.0353 (18)	0.0033 (13)	0.0022 (13)	−0.0018 (15)
C6	0.0373 (18)	0.0281 (17)	0.0368 (18)	−0.0049 (15)	0.0037 (15)	0.0009 (15)
C7	0.0304 (16)	0.0256 (16)	0.0391 (19)	−0.0055 (14)	0.0021 (14)	0.0025 (15)
C8	0.0226 (15)	0.0221 (15)	0.0303 (16)	−0.0017 (13)	0.0023 (12)	0.0017 (14)
C9	0.0236 (15)	0.0218 (15)	0.0360 (17)	−0.0023 (12)	0.0003 (13)	0.0032 (14)
C10	0.0275 (16)	0.0245 (15)	0.0347 (18)	−0.0040 (13)	0.0015 (14)	0.0027 (14)
C11	0.0376 (18)	0.0217 (15)	0.041 (2)	−0.0076 (14)	0.0032 (15)	0.0004 (15)
C12	0.0297 (16)	0.0270 (16)	0.0363 (18)	−0.0068 (14)	0.0054 (14)	0.0041 (15)
C13	0.0216 (15)	0.0259 (15)	0.0309 (17)	−0.0024 (13)	0.0034 (13)	0.0023 (14)
C14	0.0197 (14)	0.0225 (15)	0.0329 (17)	−0.0022 (12)	0.0004 (13)	0.0020 (14)
C15	0.0347 (17)	0.0219 (16)	0.0345 (18)	−0.0051 (14)	0.0065 (15)	0.0028 (14)
C16	0.0286 (16)	0.0286 (16)	0.0310 (17)	0.0015 (14)	0.0038 (13)	0.0041 (14)
C17	0.0184 (14)	0.0240 (15)	0.0367 (18)	0.0006 (13)	0.0039 (13)	0.0037 (15)
C18	0.0302 (17)	0.0318 (17)	0.0359 (18)	0.0061 (14)	0.0053 (14)	0.0028 (15)
C19	0.0386 (18)	0.0293 (17)	0.0333 (18)	0.0044 (16)	0.0028 (14)	0.0003 (15)
C20	0.0273 (16)	0.0302 (17)	0.0377 (18)	0.0043 (14)	0.0092 (14)	0.0042 (15)
C21	0.041 (2)	0.0377 (19)	0.051 (2)	−0.0022 (16)	0.0211 (17)	0.0015 (17)
C22	0.0275 (16)	0.0313 (17)	0.0297 (17)	0.0058 (14)	0.0093 (14)	0.0024 (15)
O22	0.0297 (11)	0.0341 (12)	0.0302 (11)	−0.0033 (10)	0.0061 (9)	−0.0011 (10)
C23	0.0325 (18)	0.0353 (18)	0.0340 (18)	0.0090 (15)	0.0043 (15)	0.0007 (16)
C24	0.0354 (18)	0.043 (2)	0.0316 (18)	0.0026 (16)	0.0053 (15)	−0.0024 (16)
C25	0.0391 (18)	0.0338 (18)	0.0303 (17)	−0.0016 (15)	0.0088 (15)	0.0002 (15)
C26	0.0363 (18)	0.0296 (18)	0.044 (2)	0.0050 (14)	0.0132 (16)	−0.0007 (16)
O26	0.0270 (11)	0.0319 (12)	0.0403 (13)	0.0046 (10)	0.0073 (10)	0.0001 (11)
C27	0.050 (2)	0.043 (2)	0.053 (2)	−0.0026 (18)	0.0111 (18)	−0.0088 (19)
C51	0.063 (3)	0.040 (2)	0.041 (2)	−0.0044 (19)	0.0073 (19)	−0.0030 (18)
C52	0.068 (3)	0.040 (2)	0.048 (2)	−0.010 (2)	0.014 (2)	−0.0079 (19)
C53	0.039 (2)	0.063 (3)	0.041 (2)	−0.019 (2)	0.0092 (17)	−0.003 (2)
O53	0.0594 (16)	0.087 (2)	0.0365 (15)	−0.0273 (16)	0.0049 (13)	−0.0063 (15)
C54	0.0335 (19)	0.055 (2)	0.037 (2)	0.0004 (17)	−0.0001 (16)	0.0055 (18)
C55	0.0303 (18)	0.047 (2)	0.038 (2)	−0.0033 (16)	0.0013 (15)	0.0010 (17)
C56	0.061 (2)	0.0367 (19)	0.039 (2)	0.0054 (18)	−0.0082 (18)	0.0119 (18)
C57	0.063 (2)	0.0325 (19)	0.040 (2)	0.0067 (18)	−0.0077 (18)	−0.0005 (17)
C58	0.0340 (18)	0.0296 (17)	0.0393 (19)	−0.0023 (15)	−0.0045 (15)	0.0047 (16)
C59	0.0317 (17)	0.0293 (17)	0.0409 (19)	−0.0041 (15)	−0.0016 (15)	0.0016 (16)
C60	0.0352 (18)	0.0341 (18)	0.0361 (19)	−0.0007 (15)	0.0009 (15)	0.0024 (16)
C61	0.056 (2)	0.0251 (18)	0.049 (2)	−0.0011 (16)	0.0124 (18)	−0.0003 (16)
C62	0.045 (2)	0.0276 (17)	0.048 (2)	−0.0004 (16)	0.0076 (17)	0.0036 (17)
C63	0.0244 (16)	0.0280 (16)	0.0401 (19)	−0.0008 (14)	0.0046 (14)	0.0058 (15)
C64	0.0298 (16)	0.0240 (16)	0.0386 (19)	0.0010 (14)	−0.0067 (14)	0.0022 (15)
C65	0.044 (2)	0.0308 (18)	0.0369 (19)	0.0047 (16)	−0.0083 (16)	0.0069 (16)
C66	0.0290 (17)	0.0326 (17)	0.0405 (19)	0.0017 (14)	−0.0060 (15)	−0.0038 (16)
C67	0.0227 (16)	0.0305 (18)	0.046 (2)	−0.0039 (14)	0.0008 (14)	0.0036 (16)
C68	0.0287 (17)	0.048 (2)	0.041 (2)	0.0065 (16)	0.0050 (15)	0.0124 (17)
C69	0.0361 (18)	0.053 (2)	0.043 (2)	0.0024 (17)	0.0017 (16)	0.0069 (18)
C70	0.0302 (17)	0.0338 (18)	0.043 (2)	0.0029 (15)	0.0058 (15)	0.0086 (17)
C71	0.079 (3)	0.036 (2)	0.060 (3)	−0.001 (2)	0.021 (2)	0.012 (2)
C72	0.0230 (16)	0.0364 (18)	0.0382 (19)	−0.0005 (14)	0.0039 (14)	0.0108 (16)
O72	0.0356 (12)	0.0330 (12)	0.0330 (12)	−0.0068 (10)	−0.0031 (10)	0.0020 (10)
C73	0.0271 (17)	0.060 (2)	0.038 (2)	−0.0022 (17)	−0.0009 (15)	0.0130 (19)
C74	0.0326 (18)	0.064 (2)	0.0323 (18)	−0.0096 (18)	−0.0087 (15)	0.0074 (19)
C75	0.045 (2)	0.043 (2)	0.0304 (18)	−0.0048 (17)	0.0014 (15)	0.0095 (17)
C76	0.0329 (18)	0.0352 (19)	0.0417 (19)	−0.0019 (16)	0.0030 (15)	0.0005 (17)
O76	0.0275 (11)	0.0351 (13)	0.0499 (14)	−0.0038 (10)	0.0080 (10)	−0.0017 (11)
C77	0.064 (3)	0.063 (3)	0.035 (2)	−0.007 (2)	−0.0005 (18)	0.0020 (19)

### Geometric parameters (Å, º)

**Table:**

C1—C2	1.524 (4)	C51—C52	1.525 (5)
C1—C10	1.538 (4)	C51—C60	1.539 (5)
C1—H1A	0.9900	C51—H51A	0.9900
C1—H1B	0.9900	C51—H51B	0.9900
C2—C3	1.495 (5)	C52—C53	1.497 (5)
C2—H2A	0.9900	C52—H52A	0.9900
C2—H2B	0.9900	C52—H52B	0.9900
C3—O3	1.234 (4)	C53—O53	1.229 (4)
C3—C4	1.460 (5)	C53—C54	1.455 (5)
C4—C5	1.340 (4)	C54—C55	1.346 (4)
C4—H4A	0.9500	C54—H54A	0.9500
C5—C6	1.506 (4)	C55—C56	1.507 (5)
C5—C10	1.524 (4)	C55—C60	1.515 (4)
C6—C7	1.522 (4)	C56—C57	1.511 (5)
C6—H6A	0.9900	C56—H56A	0.9900
C6—H6B	0.9900	C56—H56B	0.9900
C7—C8	1.526 (4)	C57—C58	1.522 (4)
C7—H7A	0.9900	C57—H57A	0.9900
C7—H7B	0.9900	C57—H57B	0.9900
C8—C14	1.517 (4)	C58—C64	1.521 (4)
C8—C9	1.544 (4)	C58—C59	1.548 (4)
C8—H8A	1.0000	C58—H58A	1.0000
C9—C11	1.540 (4)	C59—C61	1.540 (4)
C9—C10	1.553 (4)	C59—C60	1.562 (4)
C9—H9A	1.0000	C59—H59A	1.0000
C10—C19	1.544 (4)	C60—C69	1.549 (4)
C11—C12	1.528 (4)	C61—C62	1.529 (4)
C11—H11A	0.9900	C61—H61A	0.9900
C11—H11B	0.9900	C61—H61B	0.9900
C12—C13	1.527 (4)	C62—C63	1.525 (4)
C12—H12A	0.9900	C62—H62A	0.9900
C12—H12B	0.9900	C62—H62B	0.9900
C13—C18	1.538 (4)	C63—C68	1.531 (4)
C13—C14	1.544 (4)	C63—C64	1.543 (4)
C13—C17	1.548 (4)	C63—C67	1.552 (4)
C14—C15	1.535 (4)	C64—C65	1.533 (4)
C14—H14A	1.0000	C64—H64A	1.0000
C15—C16	1.529 (4)	C65—C66	1.531 (4)
C15—H15A	0.9900	C65—H65A	0.9900
C15—H15B	0.9900	C65—H65B	0.9900
C16—O22	1.445 (3)	C66—O72	1.448 (4)
C16—C17	1.543 (4)	C66—C67	1.547 (4)
C16—H16A	1.0000	C66—H66A	1.0000
C17—C20	1.533 (4)	C67—C70	1.532 (4)
C17—H17A	1.0000	C67—H67A	1.0000
C18—H18A	0.9800	C68—H68A	0.9800
C18—H18B	0.9800	C68—H68B	0.9800
C18—H18C	0.9800	C68—H68C	0.9800
C19—H19A	0.9800	C69—H69A	0.9800
C19—H19B	0.9800	C69—H69B	0.9800
C19—H19C	0.9800	C69—H69C	0.9800
C20—C21	1.531 (4)	C70—C71	1.526 (5)
C20—C22	1.532 (4)	C70—C72	1.534 (5)
C20—H20A	1.0000	C70—H70A	1.0000
C21—H21A	0.9800	C71—H71A	0.9800
C21—H21B	0.9800	C71—H71B	0.9800
C21—H21C	0.9800	C71—H71C	0.9800
C22—O22	1.423 (3)	C72—O72	1.421 (4)
C22—O26	1.428 (3)	C72—O76	1.428 (3)
C22—C23	1.513 (4)	C72—C73	1.510 (4)
C23—C24	1.521 (5)	C73—C74	1.529 (5)
C23—H23A	0.9900	C73—H73A	0.9900
C23—H23B	0.9900	C73—H73B	0.9900
C24—C25	1.526 (4)	C74—C75	1.508 (4)
C24—H24A	0.9900	C74—H74A	0.9900
C24—H24B	0.9900	C74—H74B	0.9900
C25—C26	1.514 (4)	C75—C77	1.518 (5)
C25—C27	1.520 (5)	C75—C76	1.522 (4)
C25—H25A	1.0000	C75—H75A	1.0000
C26—O26	1.429 (4)	C76—O76	1.432 (4)
C26—H26A	0.9900	C76—H76A	0.9900
C26—H26B	0.9900	C76—H76B	0.9900
C27—H27A	0.9800	C77—H77A	0.9800
C27—H27B	0.9800	C77—H77B	0.9800
C27—H27C	0.9800	C77—H77C	0.9800
			
C2—C1—C10	113.5 (2)	C52—C51—C60	112.9 (3)
C2—C1—H1A	108.9	C52—C51—H51A	109.0
C10—C1—H1A	108.9	C60—C51—H51A	109.0
C2—C1—H1B	108.9	C52—C51—H51B	109.0
C10—C1—H1B	108.9	C60—C51—H51B	109.0
H1A—C1—H1B	107.7	H51A—C51—H51B	107.8
C3—C2—C1	111.0 (3)	C53—C52—C51	112.0 (3)
C3—C2—H2A	109.4	C53—C52—H52A	109.2
C1—C2—H2A	109.4	C51—C52—H52A	109.2
C3—C2—H2B	109.4	C53—C52—H52B	109.2
C1—C2—H2B	109.4	C51—C52—H52B	109.2
H2A—C2—H2B	108.0	H52A—C52—H52B	107.9
O3—C3—C4	122.1 (3)	O53—C53—C54	122.0 (4)
O3—C3—C2	121.9 (3)	O53—C53—C52	122.0 (4)
C4—C3—C2	116.0 (3)	C54—C53—C52	116.0 (3)
C5—C4—C3	123.2 (3)	C55—C54—C53	123.7 (3)
C5—C4—H4A	118.4	C55—C54—H54A	118.2
C3—C4—H4A	118.4	C53—C54—H54A	118.2
C4—C5—C6	120.4 (3)	C54—C55—C56	120.9 (3)
C4—C5—C10	123.5 (3)	C54—C55—C60	123.0 (3)
C6—C5—C10	116.0 (3)	C56—C55—C60	116.0 (3)
C5—C6—C7	112.7 (2)	C55—C56—C57	112.9 (3)
C5—C6—H6A	109.1	C55—C56—H56A	109.0
C7—C6—H6A	109.1	C57—C56—H56A	109.0
C5—C6—H6B	109.1	C55—C56—H56B	109.0
C7—C6—H6B	109.1	C57—C56—H56B	109.0
H6A—C6—H6B	107.8	H56A—C56—H56B	107.8
C6—C7—C8	113.1 (2)	C56—C57—C58	112.6 (3)
C6—C7—H7A	109.0	C56—C57—H57A	109.1
C8—C7—H7A	109.0	C58—C57—H57A	109.1
C6—C7—H7B	109.0	C56—C57—H57B	109.1
C8—C7—H7B	109.0	C58—C57—H57B	109.1
H7A—C7—H7B	107.8	H57A—C57—H57B	107.8
C14—C8—C7	111.7 (2)	C64—C58—C57	112.6 (3)
C14—C8—C9	108.5 (2)	C64—C58—C59	109.8 (3)
C7—C8—C9	110.3 (2)	C57—C58—C59	108.8 (3)
C14—C8—H8A	108.7	C64—C58—H58A	108.6
C7—C8—H8A	108.7	C57—C58—H58A	108.6
C9—C8—H8A	108.7	C59—C58—H58A	108.6
C11—C9—C8	111.7 (2)	C61—C59—C58	113.2 (3)
C11—C9—C10	113.2 (2)	C61—C59—C60	113.0 (3)
C8—C9—C10	113.7 (2)	C58—C59—C60	112.6 (3)
C11—C9—H9A	105.8	C61—C59—H59A	105.7
C8—C9—H9A	105.8	C58—C59—H59A	105.7
C10—C9—H9A	105.8	C60—C59—H59A	105.7
C5—C10—C1	109.8 (2)	C55—C60—C51	110.0 (3)
C5—C10—C19	107.8 (2)	C55—C60—C69	108.3 (3)
C1—C10—C19	110.0 (3)	C51—C60—C69	109.3 (3)
C5—C10—C9	108.8 (2)	C55—C60—C59	108.6 (3)
C1—C10—C9	108.5 (2)	C51—C60—C59	108.8 (3)
C19—C10—C9	112.0 (2)	C69—C60—C59	111.8 (3)
C12—C11—C9	112.4 (2)	C62—C61—C59	113.2 (3)
C12—C11—H11A	109.1	C62—C61—H61A	108.9
C9—C11—H11A	109.1	C59—C61—H61A	108.9
C12—C11—H11B	109.1	C62—C61—H61B	108.9
C9—C11—H11B	109.1	C59—C61—H61B	108.9
H11A—C11—H11B	107.9	H61A—C61—H61B	107.7
C13—C12—C11	111.1 (2)	C63—C62—C61	111.4 (3)
C13—C12—H12A	109.4	C63—C62—H62A	109.3
C11—C12—H12A	109.4	C61—C62—H62A	109.3
C13—C12—H12B	109.4	C63—C62—H62B	109.3
C11—C12—H12B	109.4	C61—C62—H62B	109.3
H12A—C12—H12B	108.0	H62A—C62—H62B	108.0
C12—C13—C18	110.3 (2)	C62—C63—C68	109.8 (3)
C12—C13—C14	108.2 (2)	C62—C63—C64	107.8 (3)
C18—C13—C14	111.4 (2)	C68—C63—C64	111.7 (3)
C12—C13—C17	114.8 (2)	C62—C63—C67	115.9 (3)
C18—C13—C17	111.8 (2)	C68—C63—C67	110.6 (3)
C14—C13—C17	99.9 (2)	C64—C63—C67	100.6 (2)
C8—C14—C15	120.9 (2)	C58—C64—C65	119.7 (3)
C8—C14—C13	115.0 (2)	C58—C64—C63	114.0 (2)
C15—C14—C13	102.6 (2)	C65—C64—C63	103.5 (2)
C8—C14—H14A	105.7	C58—C64—H64A	106.2
C15—C14—H14A	105.7	C65—C64—H64A	106.2
C13—C14—H14A	105.7	C63—C64—H64A	106.2
C16—C15—C14	102.2 (2)	C66—C65—C64	102.2 (3)
C16—C15—H15A	111.3	C66—C65—H65A	111.3
C14—C15—H15A	111.3	C64—C65—H65A	111.3
C16—C15—H15B	111.3	C66—C65—H65B	111.3
C14—C15—H15B	111.3	C64—C65—H65B	111.3
H15A—C15—H15B	109.2	H65A—C65—H65B	109.2
O22—C16—C15	113.7 (2)	O72—C66—C65	113.3 (3)
O22—C16—C17	104.8 (2)	O72—C66—C67	105.0 (3)
C15—C16—C17	107.4 (2)	C65—C66—C67	107.8 (3)
O22—C16—H16A	110.2	O72—C66—H66A	110.2
C15—C16—H16A	110.2	C65—C66—H66A	110.2
C17—C16—H16A	110.2	C67—C66—H66A	110.2
C20—C17—C16	104.9 (2)	C70—C67—C66	104.7 (3)
C20—C17—C13	120.0 (2)	C70—C67—C63	120.3 (3)
C16—C17—C13	104.5 (2)	C66—C67—C63	104.6 (3)
C20—C17—H17A	109.0	C70—C67—H67A	108.9
C16—C17—H17A	109.0	C66—C67—H67A	108.9
C13—C17—H17A	109.0	C63—C67—H67A	108.9
C13—C18—H18A	109.5	C63—C68—H68A	109.5
C13—C18—H18B	109.5	C63—C68—H68B	109.5
H18A—C18—H18B	109.5	H68A—C68—H68B	109.5
C13—C18—H18C	109.5	C63—C68—H68C	109.5
H18A—C18—H18C	109.5	H68A—C68—H68C	109.5
H18B—C18—H18C	109.5	H68B—C68—H68C	109.5
C10—C19—H19A	109.5	C60—C69—H69A	109.5
C10—C19—H19B	109.5	C60—C69—H69B	109.5
H19A—C19—H19B	109.5	H69A—C69—H69B	109.5
C10—C19—H19C	109.5	C60—C69—H69C	109.5
H19A—C19—H19C	109.5	H69A—C69—H69C	109.5
H19B—C19—H19C	109.5	H69B—C69—H69C	109.5
C21—C20—C22	115.0 (3)	C71—C70—C67	115.0 (3)
C21—C20—C17	113.9 (3)	C71—C70—C72	115.5 (3)
C22—C20—C17	103.5 (2)	C67—C70—C72	103.8 (2)
C21—C20—H20A	108.1	C71—C70—H70A	107.3
C22—C20—H20A	108.1	C67—C70—H70A	107.3
C17—C20—H20A	108.1	C72—C70—H70A	107.3
C20—C21—H21A	109.5	C70—C71—H71A	109.5
C20—C21—H21B	109.5	C70—C71—H71B	109.5
H21A—C21—H21B	109.5	H71A—C71—H71B	109.5
C20—C21—H21C	109.5	C70—C71—H71C	109.5
H21A—C21—H21C	109.5	H71A—C71—H71C	109.5
H21B—C21—H21C	109.5	H71B—C71—H71C	109.5
O22—C22—O26	110.3 (2)	O72—C72—O76	110.2 (2)
O22—C22—C23	108.8 (2)	O72—C72—C73	108.7 (2)
O26—C22—C23	111.2 (2)	O76—C72—C73	111.3 (3)
O22—C22—C20	105.0 (2)	O72—C72—C70	104.8 (2)
O26—C22—C20	106.7 (2)	O76—C72—C70	106.9 (2)
C23—C22—C20	114.8 (3)	C73—C72—C70	114.7 (3)
C22—O22—C16	106.3 (2)	C72—O72—C66	106.6 (2)
C22—C23—C24	112.9 (3)	C72—C73—C74	113.7 (3)
C22—C23—H23A	109.0	C72—C73—H73A	108.8
C24—C23—H23A	109.0	C74—C73—H73A	108.8
C22—C23—H23B	109.0	C72—C73—H73B	108.8
C24—C23—H23B	109.0	C74—C73—H73B	108.8
H23A—C23—H23B	107.8	H73A—C73—H73B	107.7
C23—C24—C25	109.9 (3)	C75—C74—C73	110.2 (3)
C23—C24—H24A	109.7	C75—C74—H74A	109.6
C25—C24—H24A	109.7	C73—C74—H74A	109.6
C23—C24—H24B	109.7	C75—C74—H74B	109.6
C25—C24—H24B	109.7	C73—C74—H74B	109.6
H24A—C24—H24B	108.2	H74A—C74—H74B	108.1
C26—C25—C27	111.4 (3)	C74—C75—C77	113.6 (3)
C26—C25—C24	108.2 (2)	C74—C75—C76	108.0 (3)
C27—C25—C24	113.0 (3)	C77—C75—C76	111.0 (3)
C26—C25—H25A	108.1	C74—C75—H75A	108.0
C27—C25—H25A	108.1	C77—C75—H75A	108.0
C24—C25—H25A	108.1	C76—C75—H75A	108.0
O26—C26—C25	112.4 (3)	O76—C76—C75	112.0 (3)
O26—C26—H26A	109.1	O76—C76—H76A	109.2
C25—C26—H26A	109.1	C75—C76—H76A	109.2
O26—C26—H26B	109.1	O76—C76—H76B	109.2
C25—C26—H26B	109.1	C75—C76—H76B	109.2
H26A—C26—H26B	107.8	H76A—C76—H76B	107.9
C22—O26—C26	113.6 (2)	C72—O76—C76	114.0 (2)
C25—C27—H27A	109.5	C75—C77—H77A	109.5
C25—C27—H27B	109.5	C75—C77—H77B	109.5
H27A—C27—H27B	109.5	H77A—C77—H77B	109.5
C25—C27—H27C	109.5	C75—C77—H77C	109.5
H27A—C27—H27C	109.5	H77A—C77—H77C	109.5
H27B—C27—H27C	109.5	H77B—C77—H77C	109.5
			
C10—C1—C2—C3	−56.6 (4)	C60—C51—C52—C53	−55.0 (4)
C1—C2—C3—O3	−146.1 (3)	C51—C52—C53—O53	−150.9 (3)
C1—C2—C3—C4	36.8 (4)	C51—C52—C53—C54	32.2 (4)
O3—C3—C4—C5	176.5 (3)	O53—C53—C54—C55	−177.9 (3)
C2—C3—C4—C5	−6.5 (5)	C52—C53—C54—C55	−1.0 (5)
C3—C4—C5—C6	172.2 (3)	C53—C54—C55—C56	169.0 (3)
C3—C4—C5—C10	−6.2 (5)	C53—C54—C55—C60	−8.3 (5)
C4—C5—C6—C7	131.3 (3)	C54—C55—C56—C57	133.2 (3)
C10—C5—C6—C7	−50.2 (3)	C60—C55—C56—C57	−49.2 (4)
C5—C6—C7—C8	50.3 (3)	C55—C56—C57—C58	51.8 (4)
C6—C7—C8—C14	−173.3 (2)	C56—C57—C58—C64	−178.0 (3)
C6—C7—C8—C9	−52.5 (3)	C56—C57—C58—C59	−56.1 (4)
C14—C8—C9—C11	−52.9 (3)	C64—C58—C59—C61	−48.2 (3)
C7—C8—C9—C11	−175.5 (2)	C57—C58—C59—C61	−171.8 (3)
C14—C8—C9—C10	177.5 (2)	C64—C58—C59—C60	−178.0 (3)
C7—C8—C9—C10	54.9 (3)	C57—C58—C59—C60	58.4 (3)
C4—C5—C10—C1	−12.7 (4)	C54—C55—C60—C51	−14.3 (4)
C6—C5—C10—C1	168.9 (2)	C56—C55—C60—C51	168.3 (3)
C4—C5—C10—C19	107.1 (3)	C54—C55—C60—C69	105.1 (4)
C6—C5—C10—C19	−71.4 (3)	C56—C55—C60—C69	−72.3 (4)
C4—C5—C10—C9	−131.3 (3)	C54—C55—C60—C59	−133.2 (3)
C6—C5—C10—C9	50.3 (3)	C56—C55—C60—C59	49.3 (4)
C2—C1—C10—C5	43.5 (4)	C52—C51—C60—C55	45.0 (4)
C2—C1—C10—C19	−74.9 (3)	C52—C51—C60—C69	−73.8 (4)
C2—C1—C10—C9	162.3 (3)	C52—C51—C60—C59	163.9 (3)
C11—C9—C10—C5	178.6 (2)	C61—C59—C60—C55	175.9 (3)
C8—C9—C10—C5	−52.5 (3)	C58—C59—C60—C55	−54.2 (3)
C11—C9—C10—C1	59.2 (3)	C61—C59—C60—C51	56.2 (4)
C8—C9—C10—C1	−171.9 (2)	C58—C59—C60—C51	−174.0 (3)
C11—C9—C10—C19	−62.3 (3)	C61—C59—C60—C69	−64.6 (3)
C8—C9—C10—C19	66.5 (3)	C58—C59—C60—C69	65.2 (3)
C8—C9—C11—C12	54.9 (3)	C58—C59—C61—C62	49.0 (4)
C10—C9—C11—C12	−175.2 (2)	C60—C59—C61—C62	178.5 (3)
C9—C11—C12—C13	−56.5 (3)	C59—C61—C62—C63	−54.2 (4)
C11—C12—C13—C18	−66.5 (3)	C61—C62—C63—C68	−64.2 (3)
C11—C12—C13—C14	55.5 (3)	C61—C62—C63—C64	57.7 (3)
C11—C12—C13—C17	166.1 (2)	C61—C62—C63—C67	169.5 (3)
C7—C8—C14—C15	−57.8 (3)	C57—C58—C64—C65	−60.1 (4)
C9—C8—C14—C15	−179.6 (2)	C59—C58—C64—C65	178.6 (3)
C7—C8—C14—C13	178.2 (2)	C57—C58—C64—C63	176.7 (3)
C9—C8—C14—C13	56.4 (3)	C59—C58—C64—C63	55.4 (3)
C12—C13—C14—C8	−58.0 (3)	C62—C63—C64—C58	−60.4 (3)
C18—C13—C14—C8	63.4 (3)	C68—C63—C64—C58	60.4 (4)
C17—C13—C14—C8	−178.4 (2)	C67—C63—C64—C58	177.8 (2)
C12—C13—C14—C15	168.7 (2)	C62—C63—C64—C65	168.0 (3)
C18—C13—C14—C15	−69.9 (3)	C68—C63—C64—C65	−71.2 (3)
C17—C13—C14—C15	48.3 (3)	C67—C63—C64—C65	46.2 (3)
C8—C14—C15—C16	−171.8 (3)	C58—C64—C65—C66	−169.1 (3)
C13—C14—C15—C16	−42.0 (3)	C63—C64—C65—C66	−40.9 (3)
C14—C15—C16—O22	134.7 (2)	C64—C65—C66—O72	135.2 (3)
C14—C15—C16—C17	19.3 (3)	C64—C65—C66—C67	19.6 (3)
O22—C16—C17—C20	16.4 (3)	O72—C66—C67—C70	15.0 (3)
C15—C16—C17—C20	137.6 (2)	C65—C66—C67—C70	136.0 (3)
O22—C16—C17—C13	−110.8 (2)	O72—C66—C67—C63	−112.4 (3)
C15—C16—C17—C13	10.5 (3)	C65—C66—C67—C63	8.6 (3)
C12—C13—C17—C20	91.8 (3)	C62—C63—C67—C70	94.0 (3)
C18—C13—C17—C20	−34.7 (3)	C68—C63—C67—C70	−31.9 (4)
C14—C13—C17—C20	−152.7 (2)	C64—C63—C67—C70	−150.1 (3)
C12—C13—C17—C16	−151.0 (2)	C62—C63—C67—C66	−148.9 (3)
C18—C13—C17—C16	82.4 (3)	C68—C63—C67—C66	85.2 (3)
C14—C13—C17—C16	−35.6 (3)	C64—C63—C67—C66	−33.0 (3)
C16—C17—C20—C21	132.5 (3)	C66—C67—C70—C71	135.3 (3)
C13—C17—C20—C21	−110.5 (3)	C63—C67—C70—C71	−107.7 (3)
C16—C17—C20—C22	7.0 (3)	C66—C67—C70—C72	8.1 (3)
C13—C17—C20—C22	123.9 (3)	C63—C67—C70—C72	125.1 (3)
C21—C20—C22—O22	−153.4 (3)	C71—C70—C72—O72	−156.0 (3)
C17—C20—C22—O22	−28.6 (3)	C67—C70—C72—O72	−29.1 (3)
C21—C20—C22—O26	−36.4 (3)	C71—C70—C72—O76	−39.0 (4)
C17—C20—C22—O26	88.4 (3)	C67—C70—C72—O76	87.9 (3)
C21—C20—C22—C23	87.3 (3)	C71—C70—C72—C73	84.9 (4)
C17—C20—C22—C23	−147.9 (2)	C67—C70—C72—C73	−148.2 (3)
O26—C22—O22—C16	−73.8 (3)	O76—C72—O72—C66	−74.4 (3)
C23—C22—O22—C16	164.0 (2)	C73—C72—O72—C66	163.4 (2)
C20—C22—O22—C16	40.7 (3)	C70—C72—O72—C66	40.4 (3)
C15—C16—O22—C22	−152.6 (2)	C65—C66—O72—C72	−152.0 (2)
C17—C16—O22—C22	−35.7 (3)	C67—C66—O72—C72	−34.7 (3)
O22—C22—C23—C24	70.5 (3)	O72—C72—C73—C74	73.0 (3)
O26—C22—C23—C24	−51.1 (3)	O76—C72—C73—C74	−48.6 (4)
C20—C22—C23—C24	−172.3 (3)	C70—C72—C73—C74	−170.2 (3)
C22—C23—C24—C25	52.3 (3)	C72—C73—C74—C75	51.0 (4)
C23—C24—C25—C26	−54.1 (3)	C73—C74—C75—C77	−177.8 (3)
C23—C24—C25—C27	−177.9 (3)	C73—C74—C75—C76	−54.2 (4)
C27—C25—C26—O26	−176.9 (3)	C74—C75—C76—O76	59.4 (4)
C24—C25—C26—O26	58.3 (3)	C77—C75—C76—O76	−175.6 (3)
O22—C22—O26—C26	−66.5 (3)	O72—C72—O76—C76	−67.9 (3)
C23—C22—O26—C26	54.2 (3)	C73—C72—O76—C76	52.8 (3)
C20—C22—O26—C26	−180.0 (2)	C70—C72—O76—C76	178.8 (2)
C25—C26—O26—C22	−59.6 (3)	C75—C76—O76—C72	−59.8 (3)
